# Supplementary material for: Bioassay-Guided Fractionation with Antimalarial and Antimicrobial Activities of Paeonia officinalis
Source: Molecules. 2022 Dec 1;27(23):8382. doi: 10.3390/molecules27238382 (PMC9739537; doi:10.3390/molecules27238382)
Supplement: Supplementary file 1 [file molecules-27-08382-s001.zip › molecules-2055515-supplementary.pdf]

Supplementary materials

Communication

Bioassay-Guided Fractionation with Antimalarial and Antimicrobial Activities of *Paeonia officinalis*

Mamdouh Nabil Samy [1,2,†], Basma Khalaf Mahmoud [1,†], Nourhan Hisham Shady [3,†], Usama Ramadan Abdelmohsen [1,3,*] and Samir Anis Ross [2,4,*]

[1] Department of Pharmacognosy, Faculty of Pharmacy, Minia University, Minia 61519, Egypt
[2] National Center for Natural Products Research, School of Pharmacy, University of Mississippi, Oxford, MS 38677, USA
[3] Department of Pharmacognosy, Faculty of Pharmacy, Deraya University, New Minia 61111, Egypt
[4] Department of BioMolecular Science, Division of Phamacognosy, School of Pharmacy, University of Mississippi, Oxford, MS 38677, USA
* Correspondence: usama.ramadan@mu.edu.eg (U.R.A.); sross@olemiss.edu (S.A.R.)
† These authors contributed equally to this work.

Abstract: Bioassay-guided fractionation technique of roots of *Paeonia officinalis* led to isolation and structure elucidation of seven known compounds, including four monoterpene glycosides: lactiflorin (**1**), paeoniflorin (**4**), galloyl paeoniflorin (**5**), and (*Z*)-(1*S*,5*R*)-*β*-pinen-10-yl *β*-vicianoside (**7**), two phenolics; benzoic acid (**2**) and methyl gallate (**3**), and one sterol glycoside; *β*-sitosterol 3-*O*-*β*-D-glucopyranoside (**6**). The different fractions and the isolated compounds were evaluated for their antimicrobial and antimalarial activities. Fraction II and III showed antifungal activity against *Candida neoformans* with IC_{50} value of 28.11 and 74.37 µg/mL, respectively, comparing with the standard fluconazole (IC_{50} = 4.68 µg/mL), and antibacterial potential against *Pseudomonas aeruginosa* (IC_{50} = 20.27 and 24.82 µg/mL, respectively), *Klebsiella pneumoniae* (IC_{50} = 43.21 and 94.4 µg/mL, respectively), comparing with the standard meropenem (IC_{50} = 28.67 and 43.94 µg/mL, respectively). Compounds 3 and 5 showed antimalarial activity against *Plasmodium falciparum* D6 with IC_{50} value of 1.57 and 4.72 µg/mL and *P. falciparum* W2 with IC_{50} value of 0.61 and 2.91 µg/mL, respectively, comparing with the standard chloroquine (IC_{50} = 0.026 and 0.14 µg/mL, respectively).

Keywords: *Paeonia officinalis*; phytoconstituents; antimalarial; antimicrobial.

Lactiflorin (**1**): ^{1}H NMR (400 MHz, CD_3OD) : 8.03 (2H, d, J =7.2 Hz, H-2′,H-6′), 7.66 (lH, t, J =7.5 Hz, H-4′), 7.54 (2H, t, *J*=7.5 Hz, H-3′,H-S), 4,92 (1H, d, *J*=4.5 Hz, H-9), 4.80 (2H, s, H-8), 4.64 (1H, d, J=8.0 Hz, H-l″), 3.87 (2H,br d, J=11.4 Hz, H-6″), 3.68 (1H, m, H-5″), 3.53(lH, td, *J*=4.45, 7.4,9.3 Hz, H-3″), 3.37 (lH, td,*J*=4.35, 7.4,9.6 Hz, H-4″), 3.23 (1H,dd,*J*=8.1,9.3Hz,H-2″), 2.82 (lH, d, *J*=17.8 Hz, H-2b), 2.78 (lH, dt, *J*=4.5,4.5, 13.4 Hz, H-4), 2.55 (1H, t, *J*= 13.4 Hz, H-5b), 2.51 (1H, d, *J*=17.8 Hz,H-2a), 2.19 (lH, dd, *J*=4.7, 14.0 Hz, 5H-a), 1.41 (3H, s, Me-10). ^{13}C NMR (100 MHz, CD_3OD); 48.2 (C-1), 86.7 (C-2), 38.7 (C-3), 81.7 (C-4), 31.6 (C-5), 104.1 (C-6), 56.8 (C-7), 63.8 (C-8), 219.3 (C-3), 16.3 (C-10), glucopyranosyl moiety; 96.4 (C-1′), 74.6 (C-2′), 80.0 (C-3′), 71.5 (C-4′), 76.0 (C-5′), 62.2 (C-6′), benzoyl moiety; 130.7 (C-1′′), 130.6 (C-2′′,6′′), 129.9 (C-3′′,5′′), 134.9 (C-4′′), 167.8 (C-7′′). HR-ESI-MS m/z 485.1431 $[M + Na]^+$ calc. 485.1424 for $C_{23}H_{26}O_{10}Na$.

Benzoic acid (2) ^{1}H NMR (400 MHz, CD_3OD); 8.04 (2H, d, *J* =7.5 Hz, H-2,H-6), 7.58 (1H, t, *J* =6.9 Hz, H-4), 7.46 (2H, t, *J* =7.5 Hz, H-3,H-5). ^{13}C NMR (100 MHz, CD_3OD) 131.8 (C-1), 130.6 (C-2, C-6), 129.4 (C-3,C-5), 134.0 (C-4). HR-ESI-MS *m/z:* 121.0282 $[M-H]^-$ calc. 121.0290 for $C_7H_5O_2$.

Methyl gallate (3) ^{1}H NMR (400 MHz, CD_3OD); 7.08 (2H, s, H-2,H-6), 3.82 (3H, s, OCH_3). ^{13}C NMR (100 MHz, CD_3OD) 121.3 (C-1), 110.2 (C-2, C-6), 146.2 (C-3,C-5), 139.7 (C-4), 169.2 (C=O), 52.5 (OCH_3). HR-ESI-MS *m/z:* 183.0361 $[M-H]^-$ calc. 183.0293 for $C_8H_7O_5$.

Paeoniflorin (**4**): ^{1}H NMR (400 MHz, CD_3OD); 1.73 (1H, d, *J* =11.8 Hz, H-3a), 1.92 (1H, d, *J* =11.8 Hz, H-3b), 2.55 (1H, d, *J* =6.5 Hz, H-5), 1.78 (1H, d, *J* =10.6 Hz, H-7a), 2.46 (1H, dd, *J* =10.6, 6.8 Hz, H-7b), 5.43 (1H, s, H-9), 1.27 (3H, s, H-10), 4.58 (1H, d, *J* =7.8 Hz, H-1'), 8.07 (2H, d, *J* =7.2 Hz, H-2'',H-6''), 7.50 (2H, t, *J* =7.6 Hz, H-3'',H-5''), 7.63 (1H, t, *J* =7.4 Hz, H-4''). ^{13}C NMR (100 MHz, CD_3OD): 89.4 (C-1), 87.4 (C-2), 44.6 (C-3), 106.5 (C-4), 44.0 (C-5), 71.8 (C-6), 23.5 (C-7), 61.8 (C-8), 102.4 (C-9), 19.7 (C-10), glucopyranosyl moiety; 100.2 (C-1'), 75.1 (C-2'), 78.0 (C-3'), 72.3 (C-4'), 78.1 (C-5'), 62.9 (C-6'), benzoyl moiety; 131.2 (C-1''), 130.8 (C-2'', C-6''), 129.8 (C-3'', C-5''), 134.6 (C-4''), 168.2 (C-7''). HR-ESI-MS *m/z:* 503.1516 $[M+Na]^+$ calc. 503.1529 for $C_{23}H_{28}O_{11}Na$.

Galloyl paeoniflorin (**5**): ^{1}H NMR (400 MHz, CD_3OD) 1.83 (1H, d, *J* =12.5 Hz, H-3a), 2.20 (1H, d, *J* =12.5 Hz, H-3b), 2.60 (1H, d, *J* =6.2 Hz, H-5), 1.96 (1H, d, *J* =10.8 Hz, H-7a), 2.51 (1H, dd, *J* =10.8, 6.8 Hz, H-7b), 5.45 (1H, s, H-9), 1.39 (3H, s, H-10), 4.54 (1H, d, *J* =7.6 Hz, H-1'), 8.01 (2H, d, *J* =7.2 Hz, H-2'',H-6''), 7.46 (2H, t, *J* =7.6 Hz, H-3'',H-5''), 7.59 (1H, t, *J* =7.4 Hz, H-4''), 7.12 (2H, s, H-2''',6'''). ^{13}C NMR (100 MHz, CD_3OD): 89.3 (C-1), 87.3 (C-2), 44.4 (C-3), 106.4 (C-4), 43.8 (C-5), 71.9 (C-6), 23.0 (C-7), 61.7 (C-8), 102.2 (C-9), 19.6 (C-10), glucopyranosyl moiety; 100.0 (C-1'), 74.9 (C-2'), 77.8 (C-3'), 72.1 (C-4'), 75.1 (C-5'), 64.7 (C-6'), benzoyl moiety; 131.0 (C-1''), 130.7 (C-2'', C-6''), 129.7 (C-3'', C-5'', 134.5 (C-4''), 168.2 (C-7''), galloyl moiety; 121.5 (C-1'''), 110.3 (C-2''', C-6'''), 146.6 (C-3''', C-5'''), 139.9 (C-4'''), 168.2 (C-7'''). HR-ESI-MS *m/z:* 655.1666 $[M+Na]^+$ calc. 655.1639 for $C_{30}H_{32}O_{15}Na$.

***β*-sitosterol 3-*O*-*β*-D-glucopyranoside** (**6**) ^{1}H NMR (C_5D_5N); 4.25 (1H, m, H-3), 5.33 (1H, br s, H-6), 0.65 (3H, s, H-18), 0.92 (3H, s, H-19), 0.97 (3H, d, *J* =6.4 Hz, H-21), 0.88 (3H, d, *J* =7.6 Hz, H-26), 0.86 (3H, d, *J* =7.0 Hz, H-27), 5.01(1H, d, *J* =7.7 Hz, H-1'). ^{13}C NMR (100 MHz, C_5D_5N); 37.3 (C-1), 30.0 (C-2), 78.4 (C-3), 39.1 (C-4), 140.7 (C-5), 121.7 (C-6), 32 (C-7), 31.9 (C-8), 50.1 (C-9), 36.7 (C-10), 21.1 (C-11), 39.8 (C-12), 42.3 (C-13), 56.6 (C-14), 24.3 (C-15), 28.3 (C-16), 56.1 (C-17), 11.8 (C-18), 19.0 (C-19), 36.2 (C-20), 18.8 (C-21), 34.0 (C-22), 26.2 (C-23), 45.8 (C-24), 29.3 (C-25), 19.2 (C-26), 19.8 (C-27), 23.2 (C-28), 12.0 (C-29), glucopyranosyl moiety; 102.4 (C-1'), 75.1 (C-2'), 78.2 (C-3'), 71.4 (C-4'), 77.9 (C-5'), 62.6 (C-6').

(*Z*)-(1*S*,5*R*)-*β*-pinen-10-yl *β*-vicianoside (**7**): ^{1}H NMR (400 MHz, CD_3OD) 3.05 (1H, t, *J* =5.5 Hz, H-1), 1.45 (2H, m, H-3), 1.27 (2H, m, H-4), 1.94 (1H, m, H-5), 2.31 (2H, m, H-7), 1.27 (3H, s, H-8), 0.78 (3H, s, H-9), 6.21(1H, s, H-10), 4.37 (1H, d, *J* =7.8 Hz, H-1'), 4.04 (1H, dd, *J* =11.5, 2.2 Hz, H-6'a), 3.76 (1H, dd, *J* =11.5, 6.6 Hz, H-6'a), 4.34 (1H, d, *J* =6.8 Hz, H-1''), 3.87 (1H, dd, *J* =12.4, 3.1 Hz, H-5''). ^{13}C NMR (100 MHz, CD_3OD): 43.4 (C-1), 123.6 (C-2), 24.8 (C-3), 20.3 (C-4), 42.1 (C-5), 43.8 (C-6), 26.7 (C-7), 22.3 (C-8), 26.6 (C-9), 137.5 (C-10), glucopyranosyl moiety; 104.2 (C-1'), 74.2 (C-2'), 77.9 (C-3'), 71.4 (C-4'), 74.7 (C-5'), 69.5 (C-6'), arabinopyranosyl moiety; 105.2 (C-1''), 72.5 (C-2''), 77.1 (C-3''), 69.7 (C-4''), 66.9 (C-5''). HR-ESI-MS *m/z:* 469.2040 $[M+Na]^+$ calc. 469.2050 for $C_{21}H_{34}O_{10}Na$.

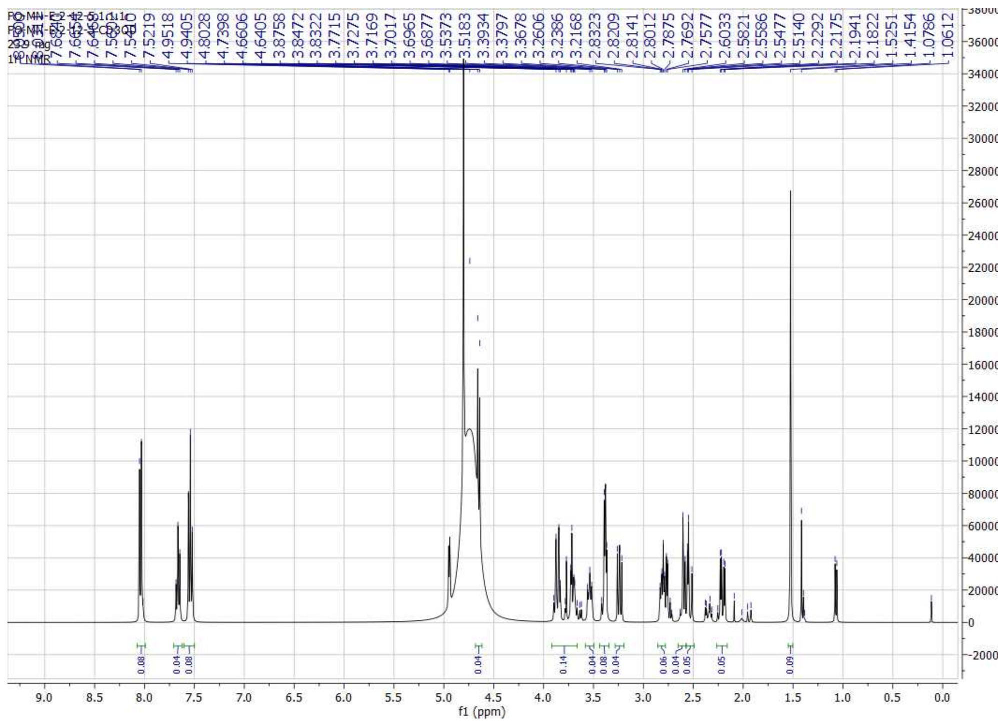

Figure S1. ^{1}H NMR spectrum of compound 1 (400 MHz, CD_3OD).

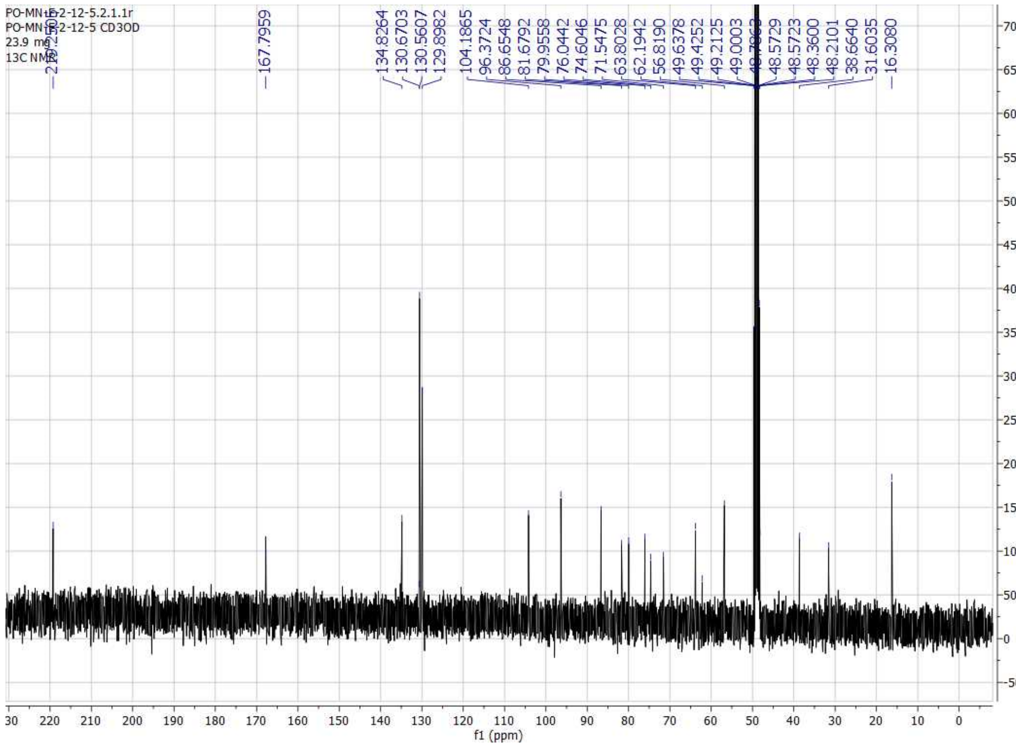

Figure S2. ^{13}C NMR spectrum of compound 1 (100 MHz, CD_3OD).

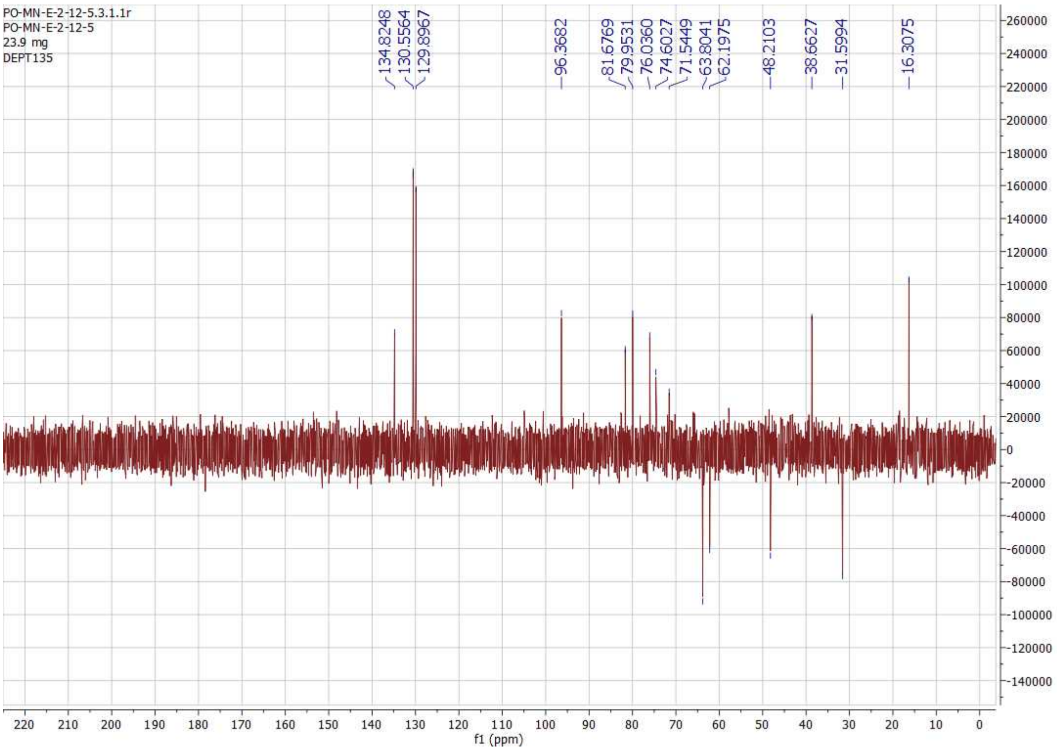

Figure S3. DEPT NMR spectrum of compound 1 (100 MHz, CD_3OD).

Compound 1 *m/z:* 485.1431 $[M+Na]^+$ calc. 485.1424 for $C_{23}H_{26}O_{10}Na$

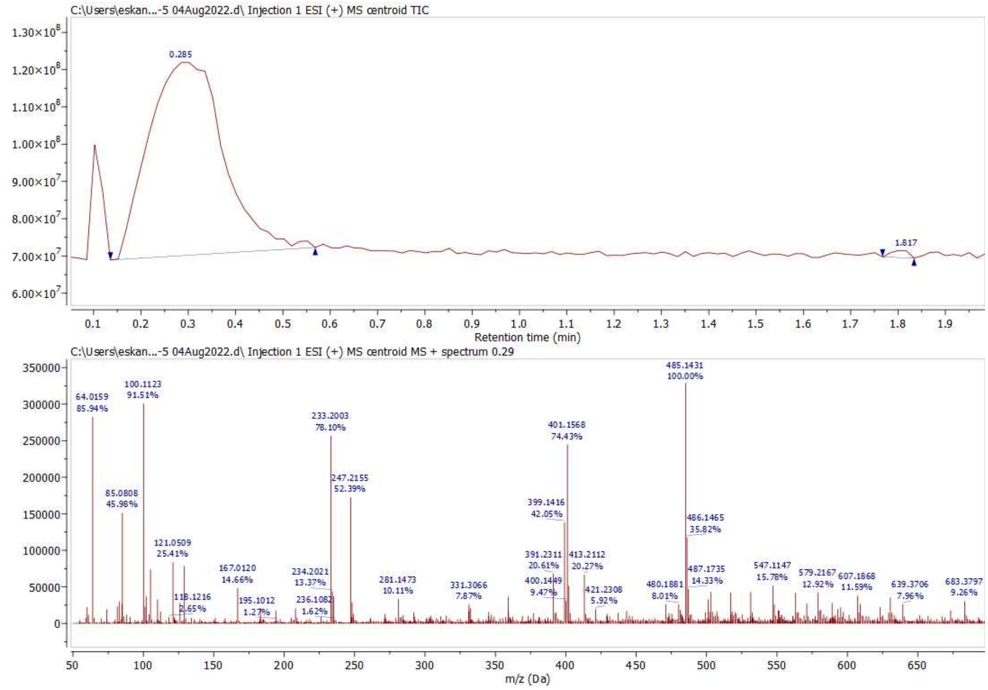

Figure S4. HR-ESI-MS spectrum of compound 1.

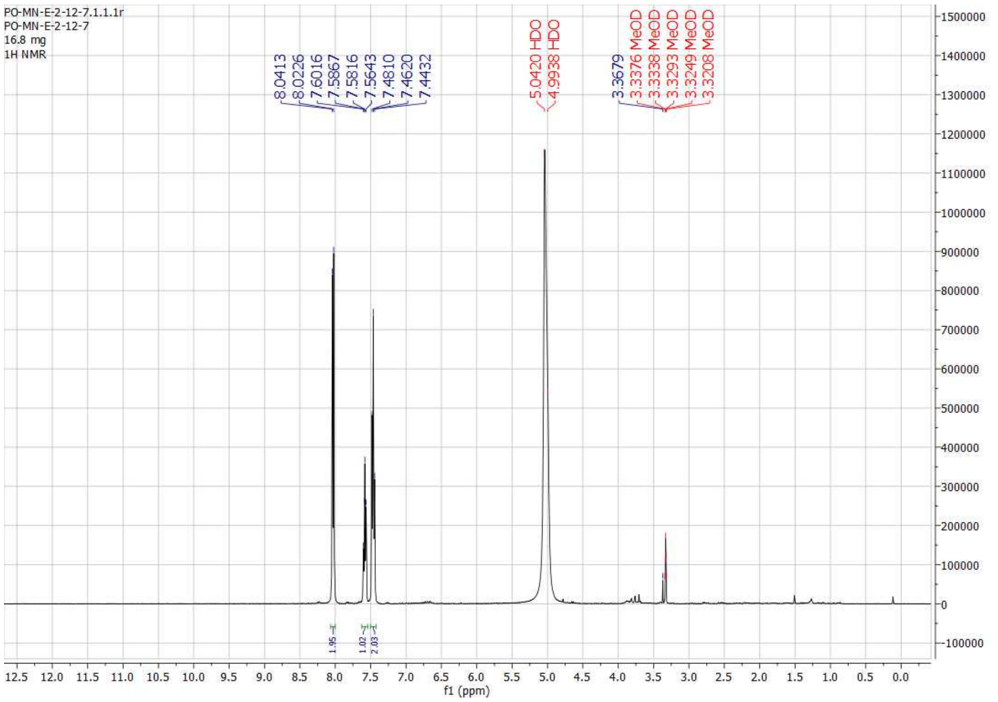

Figure S5. ^{1}H NMR spectrum of compound 2 (400 MHz, CD_3OD).

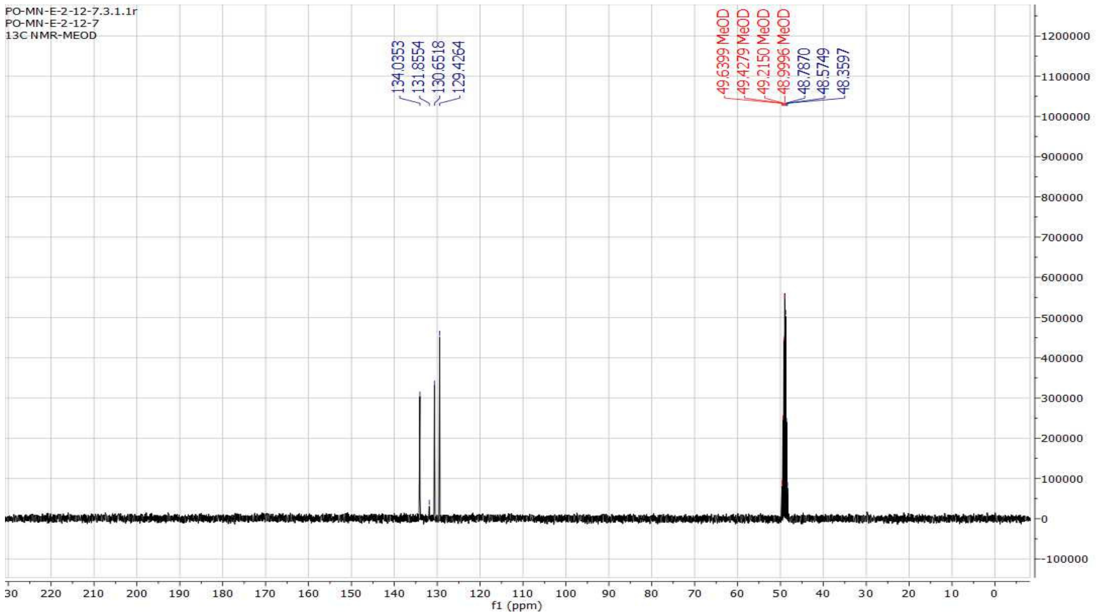

Figure S6. ^{13}C NMR spectrum of compound 2 (100 MHz, CD_3OD).

Compound 2 *m/z:* 121.0282 [M-H]$^-$ calc. 121.0290 for $C_7H_5O_2$

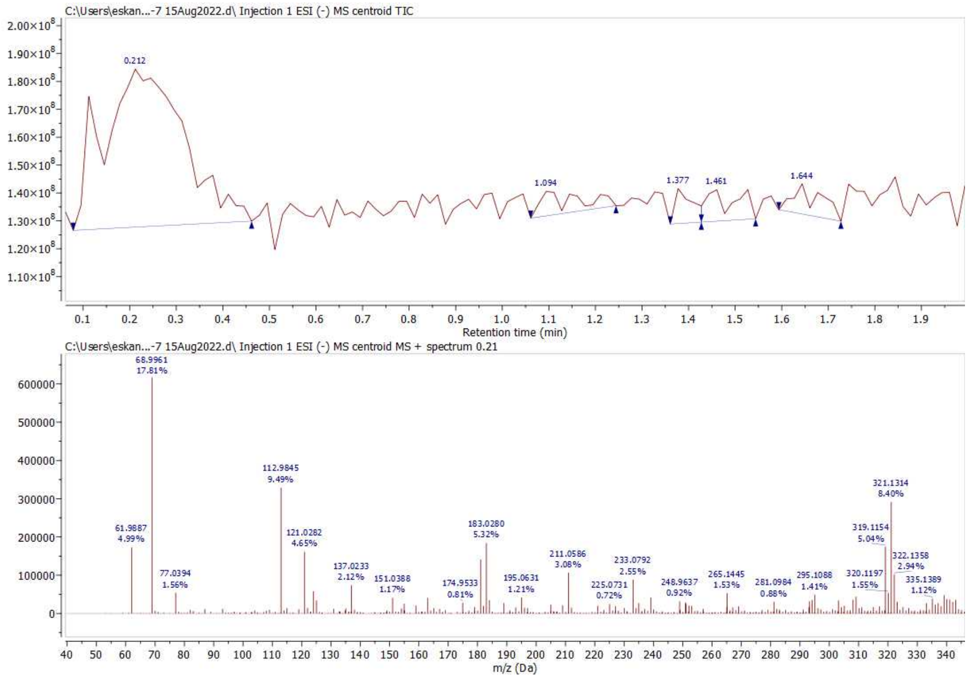

Figure S7. HR-ESI-MS spectrum of compound 2.

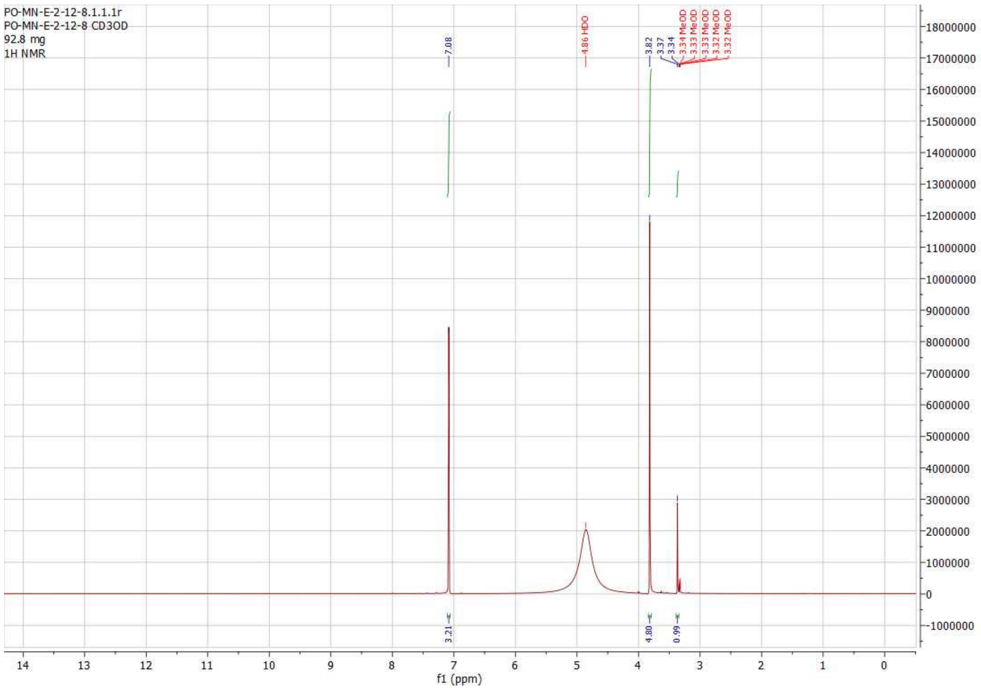

Figure S8. ^{1}H NMR spectrum of compound 3 (400 MHz, CD_3OD).

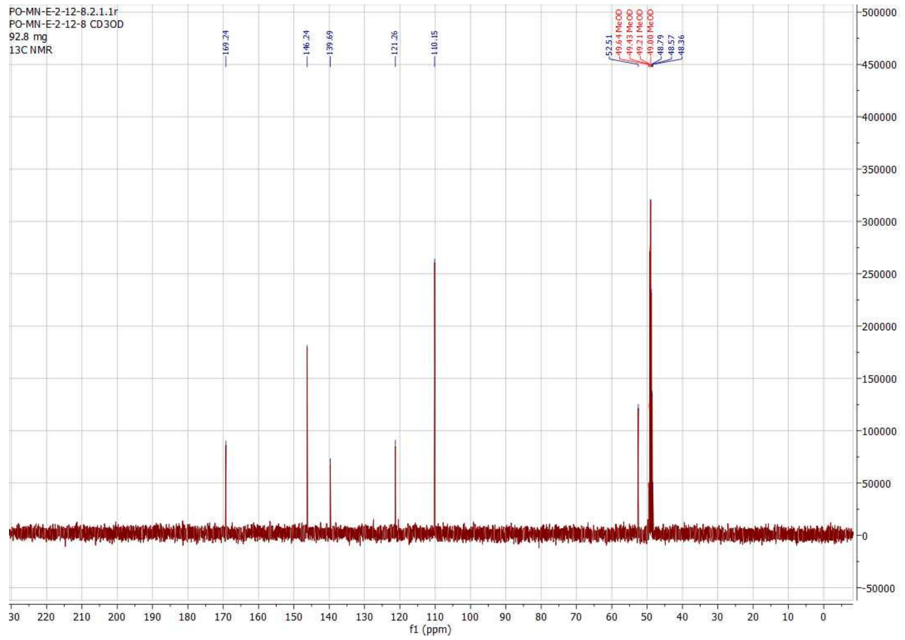

Figure S9. ^{13}C NMR spectrum of compound 3 (100 MHz, CD_3OD).

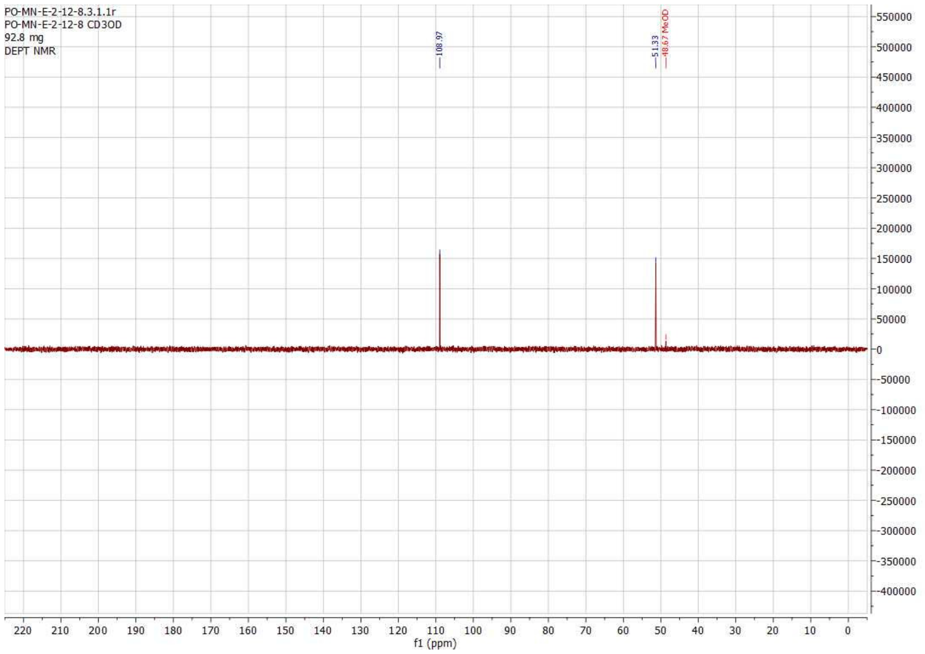

Figure S10. DEPT NMR spectrum of compound 3 (100 MHz, CD_3OD).

Compound 3 *m/z:* 183.0361 [M-H]⁻ calc. 183.0293 for $C_8H_7O_5$

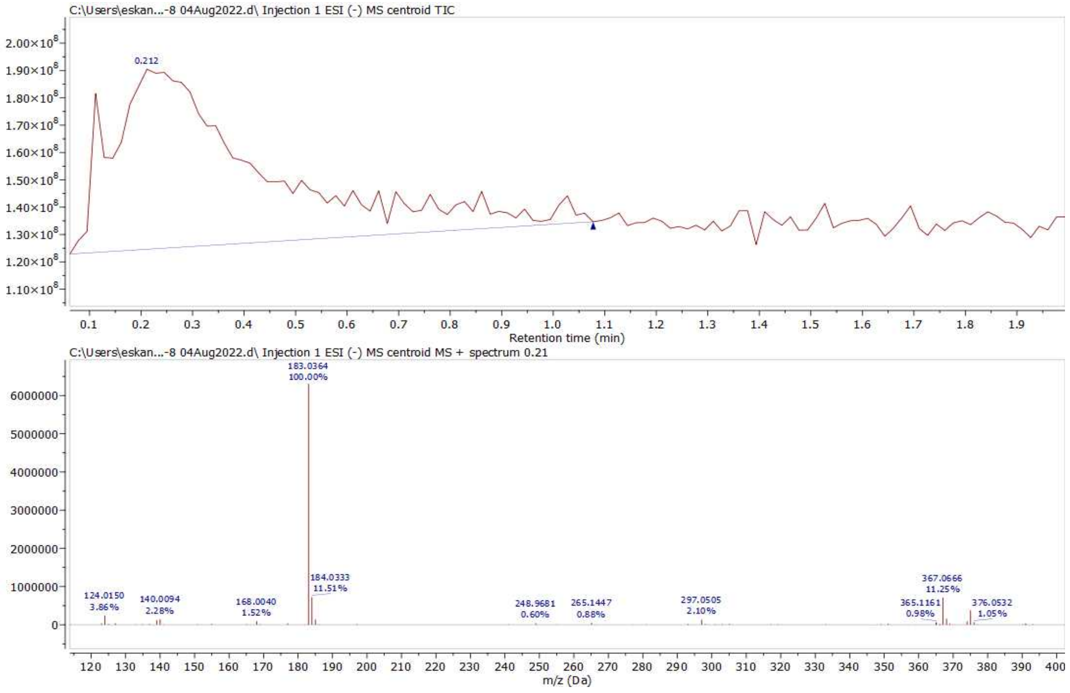

Figure S11. HR-ESI-MS spectrum of compound 3.

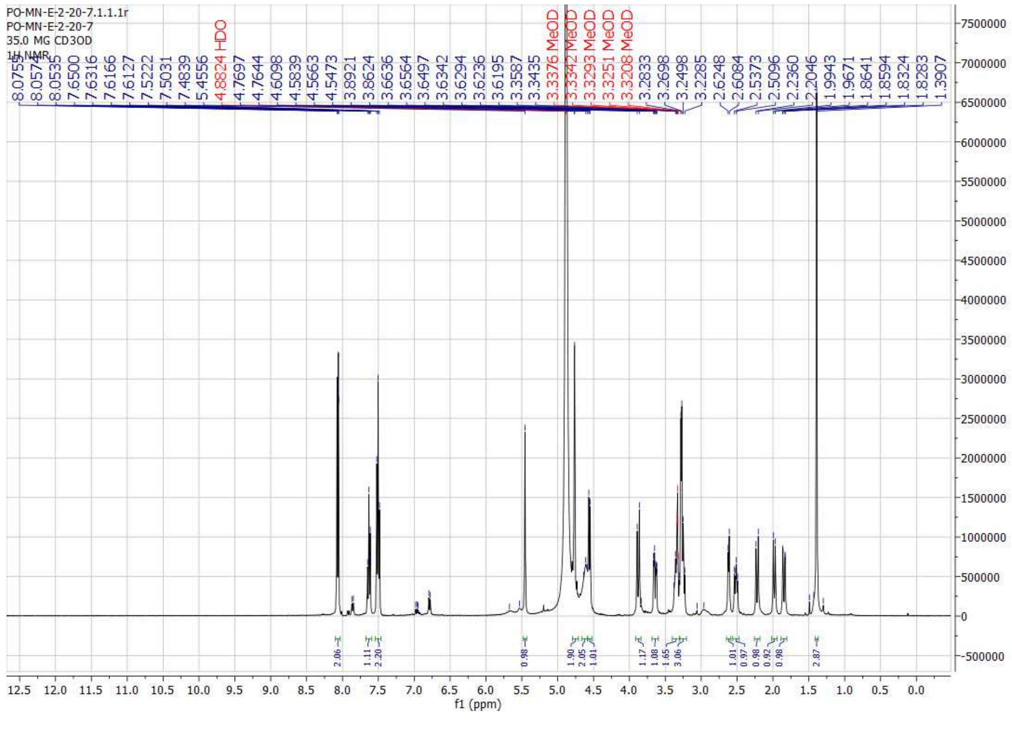

Figure S12. ^{1}H NMR spectrum of compound 4 (400 MHz, CD_3OD).

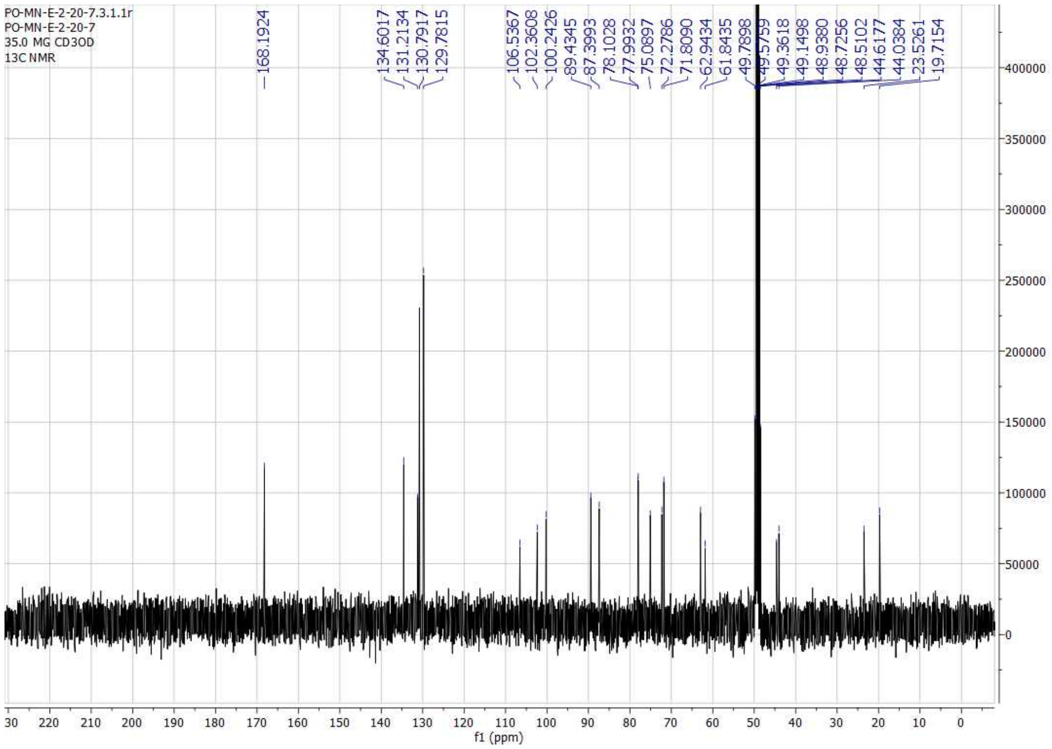

Figure S13. ^{13}C NMR spectrum of compound 4 (100 MHz, CD_3OD).

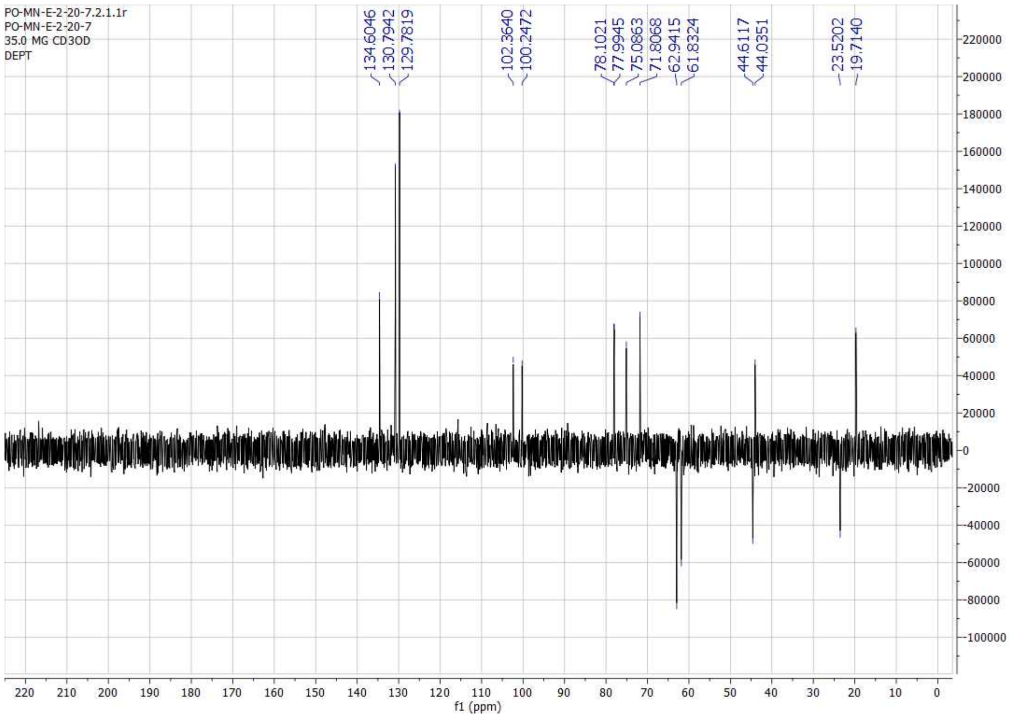

Figure S14. DEPT NMR spectrum of compound 4 (100 MHz, CD_3OD).

Compound 4 *m/z:* 503.1516 $[M+Na]^+$ calc. 503.1529 for $C_{23}H_{28}O_{11}Na$

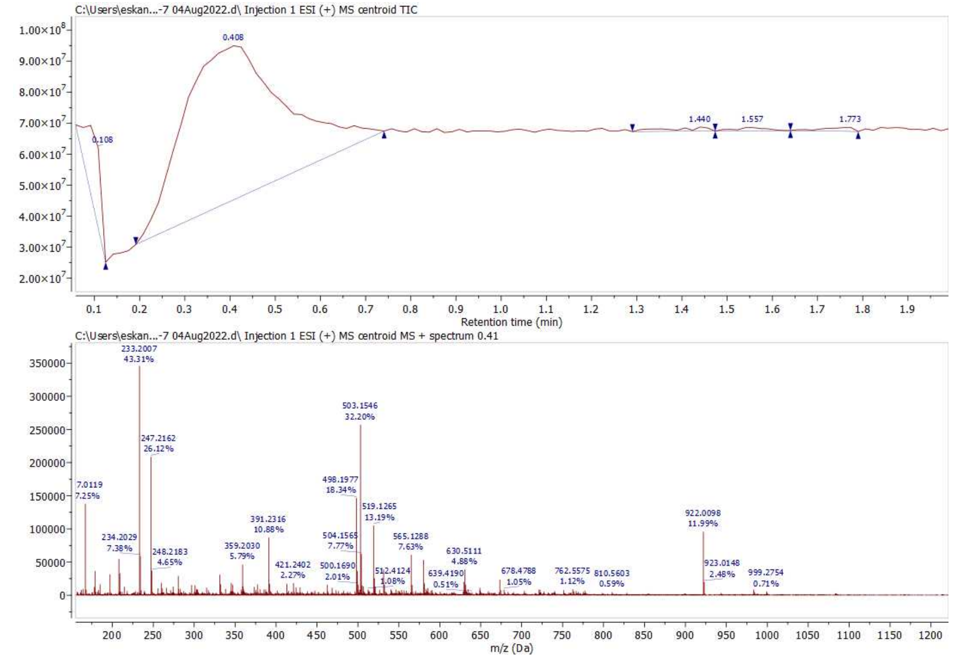

Figure S15. HR-ESI-MS spectrum of compound 4.

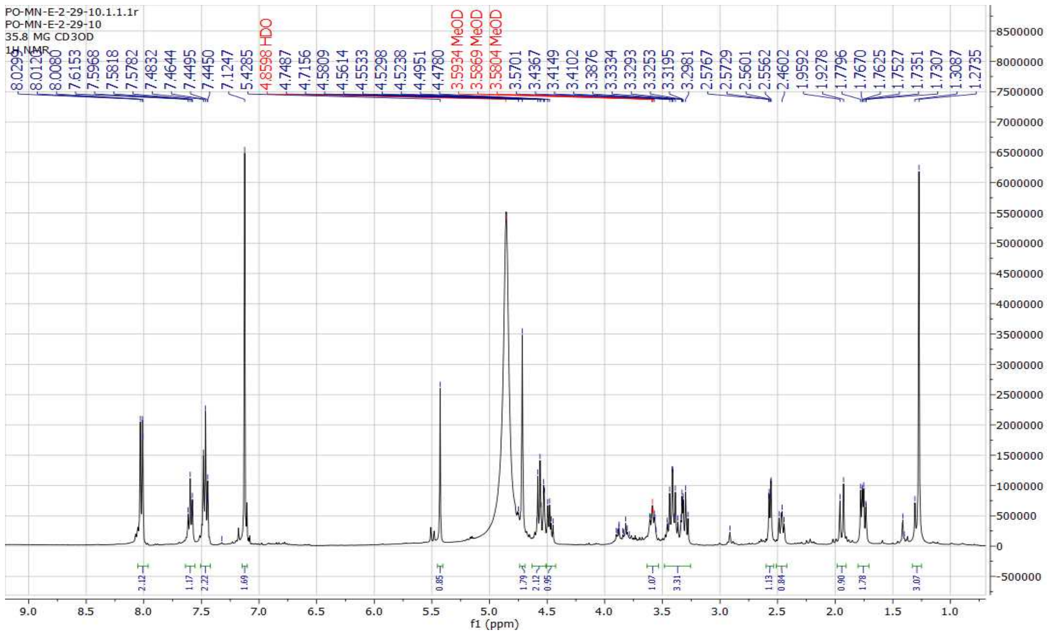

Figure S16. ^{1}H NMR spectrum of compound 5 (400 MHz, CD_3OD).

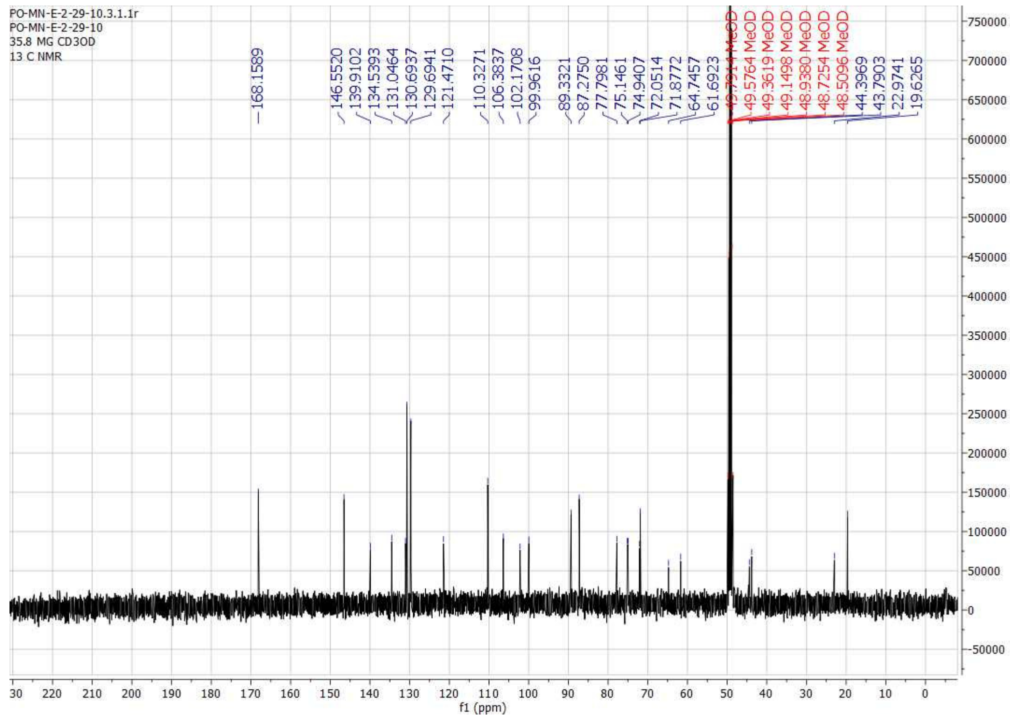

Figure S17. ^{13}C NMR spectrum of compound 5 (100 MHz, CD_3OD).

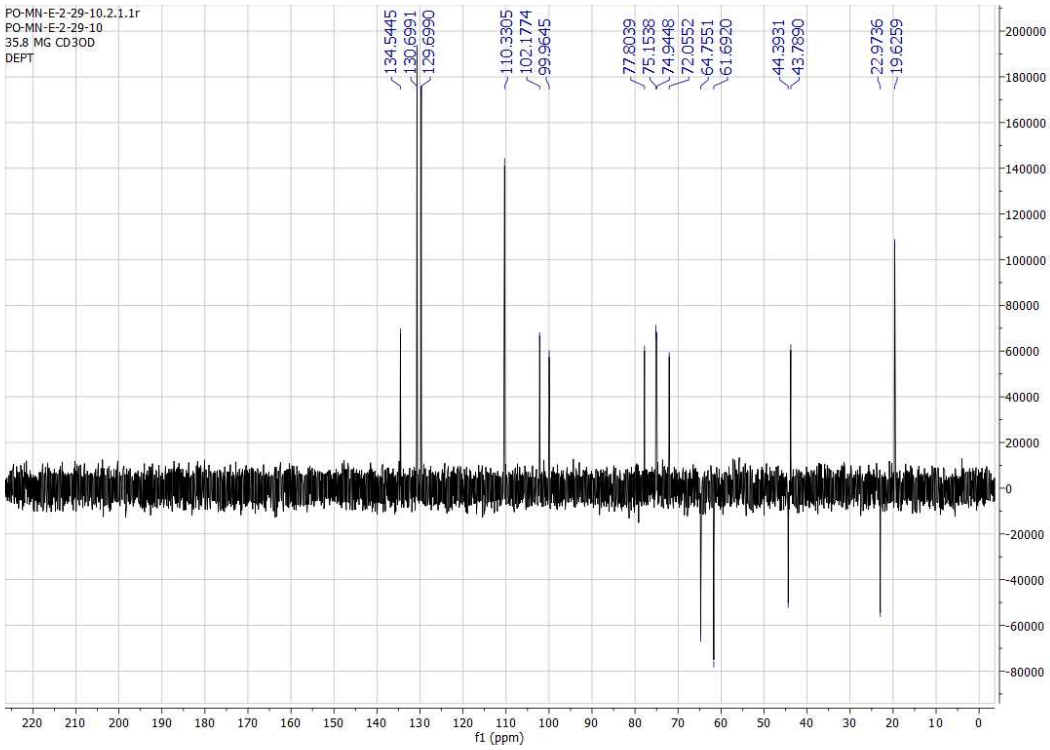

Figure S18. DEPT NMR spectrum of compound 5 (100 MHz, CD_3OD).

Compound 5 *m/z:* 655.1666 [M+Na]$^+$ calc. 655.1639 for $C_{30}H_{32}O_{15}Na$

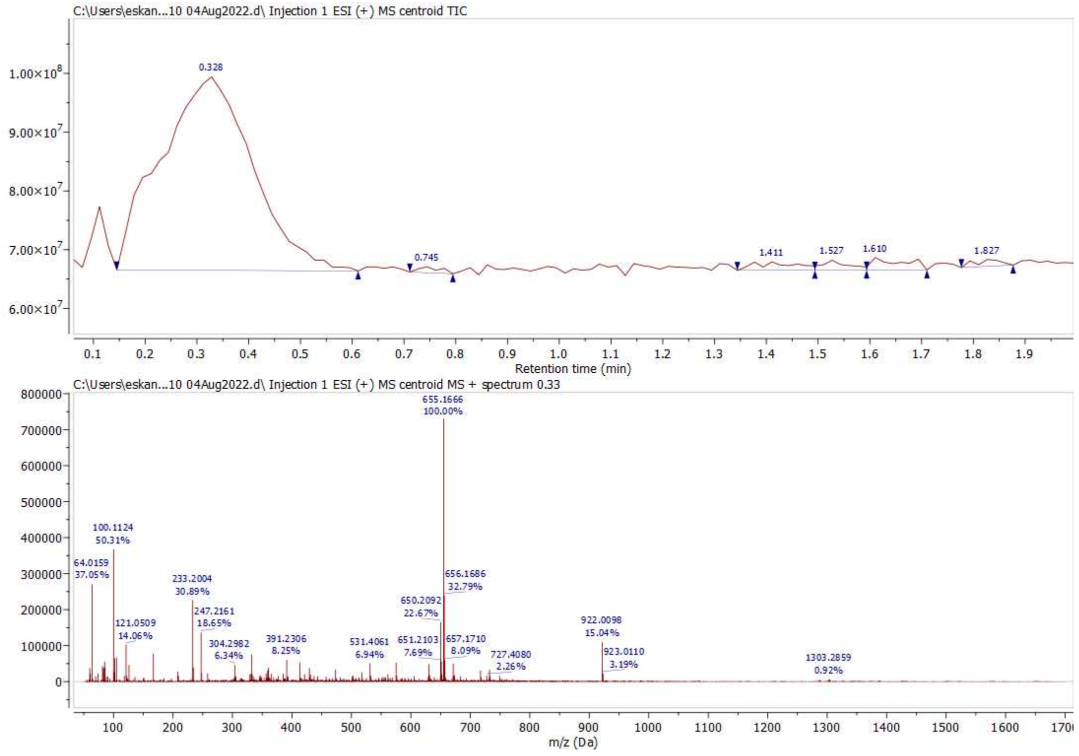

Figure S19. HR-ESI-MS spectrum of compound 5.

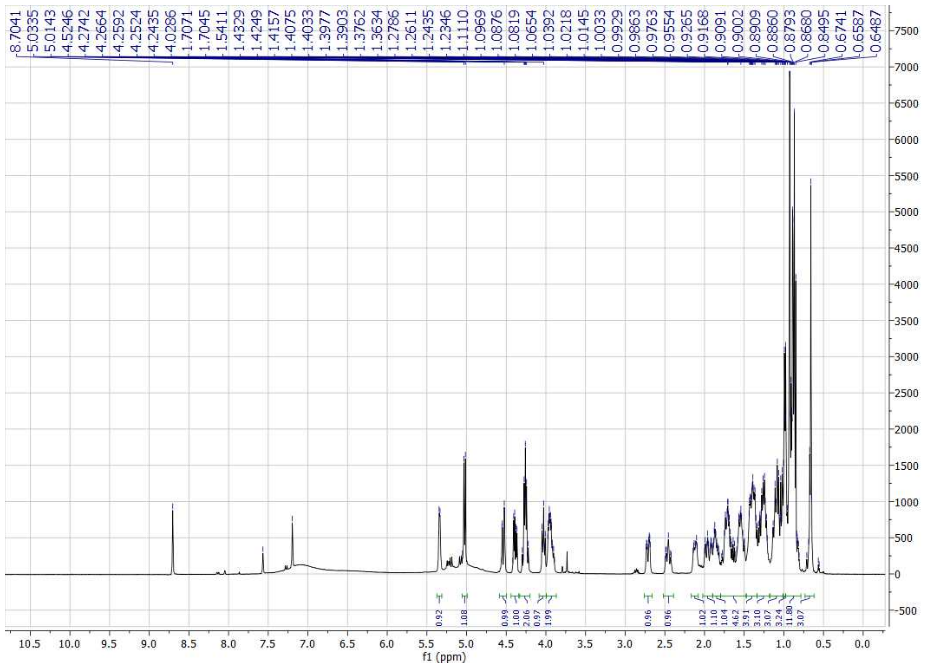

Figure S20. ^{1}H NMR spectrum of compound 6 (400 MHz, C_5D_5N).

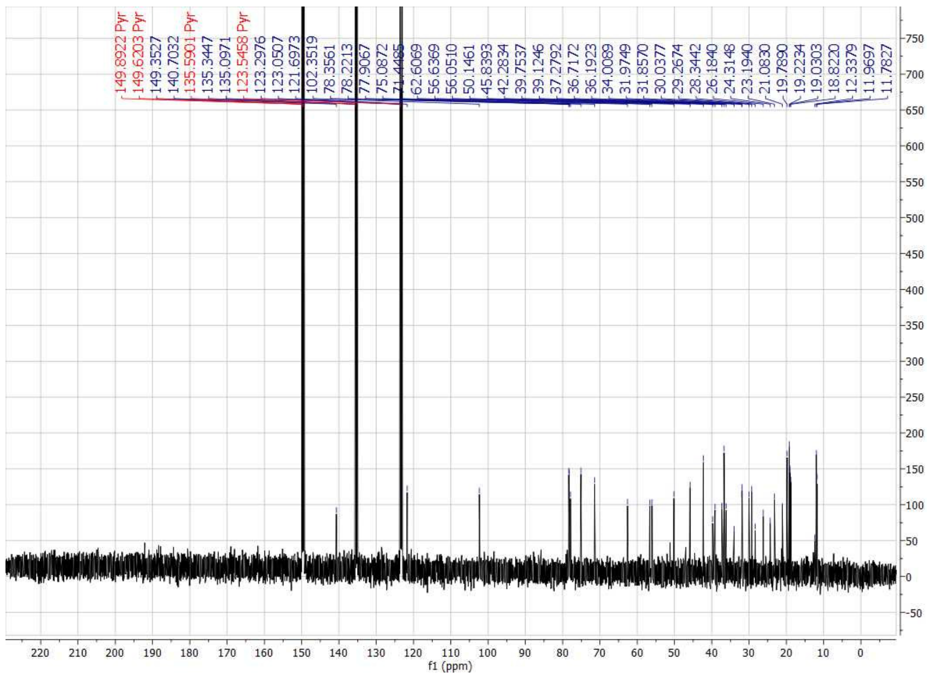

Figure S21. ^{13}C NMR spectrum of compound 6 (400 MHz, C_5D_5N).

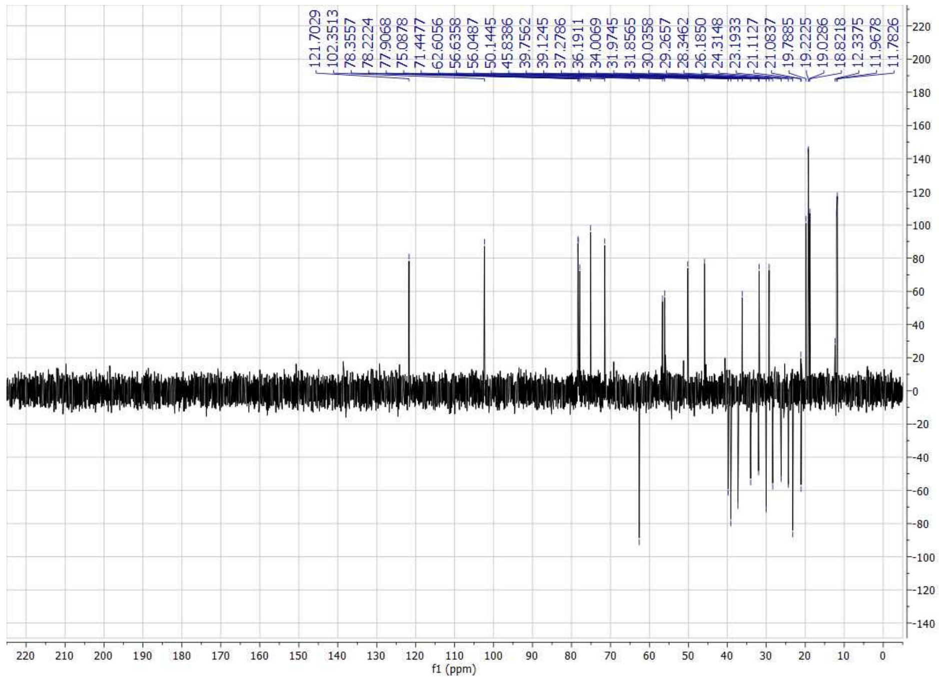

Figure S22. DEPT NMR spectrum of compound 6 (400 MHz, C_5D_5N).

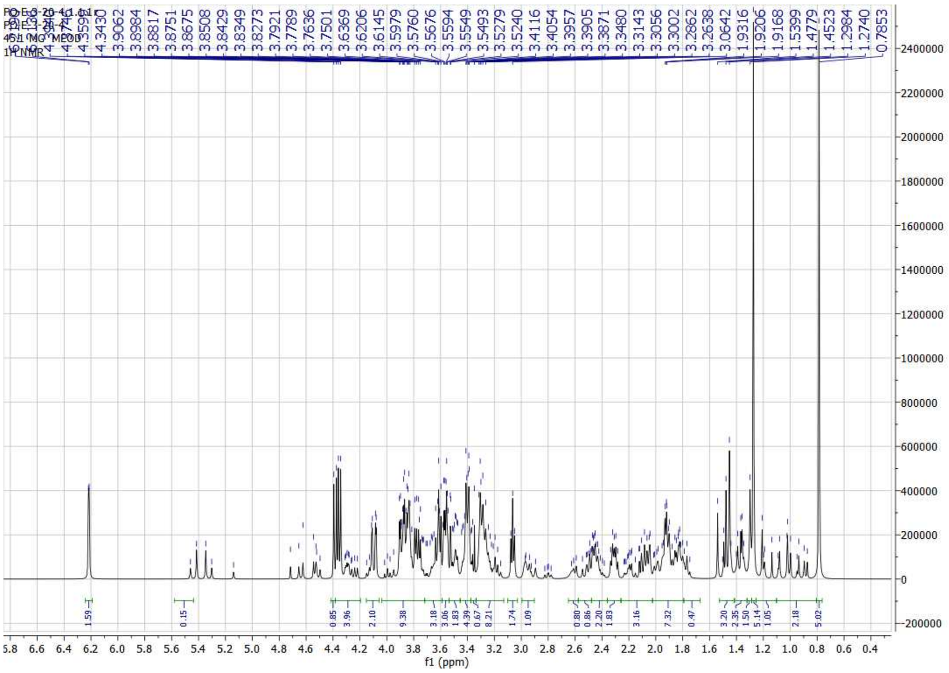

Figure S23. ^{1}H NMR spectrum of compound 7 (400 MHz, CD_3OD).

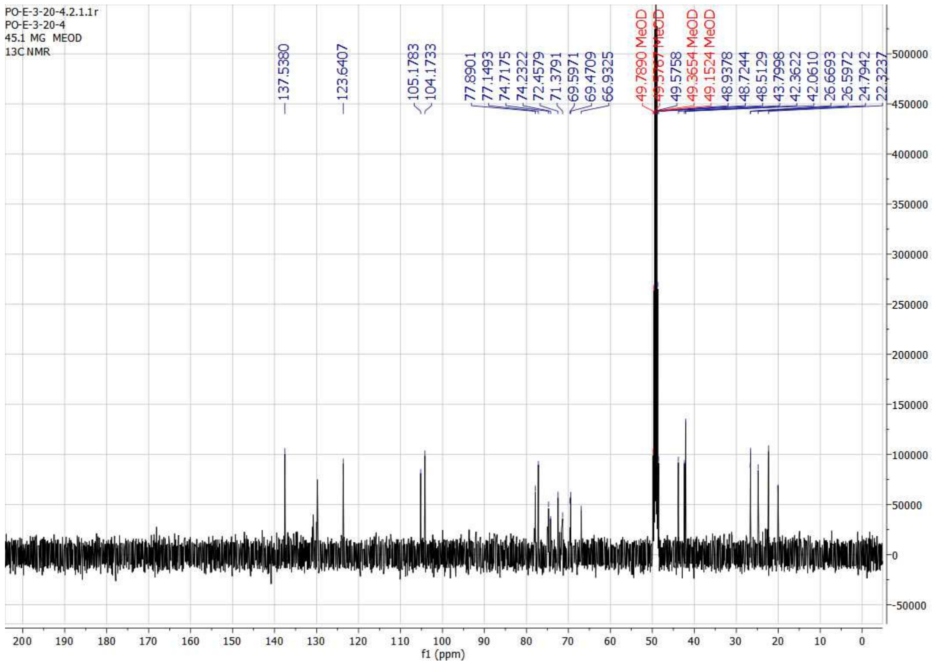

Figure S24. ^{13}C NMR spectrum of compound 7 (100 MHz, CD_3OD).

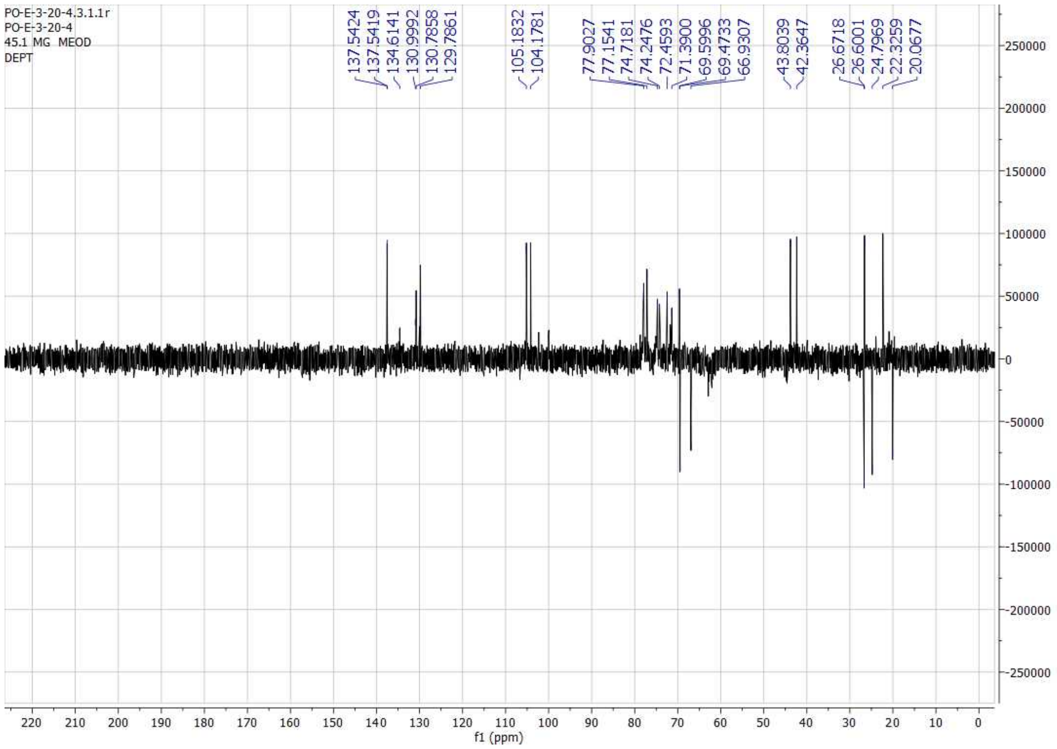

Figure S25. DEPT NMR spectrum of compound 7 (100 MHz, CD_3OD).

Compound 7 *m/z:* 469.2040 $[M+Na]^+$ calc. 469.2050 for $C_{21}H_{34}O_{10}Na$

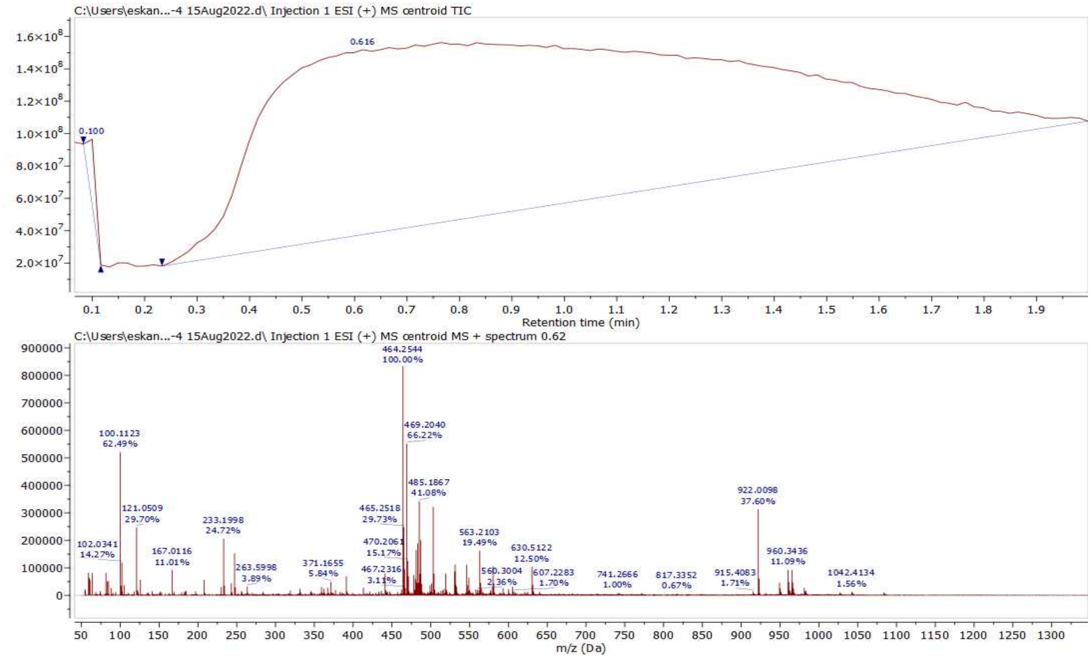

Figure S26. HR-ESI-MS spectrum of compound 7.
